# Peak strain magnitudes and rates in the tibia exceed greatly those in the skull: An in vivo study in a human subject

**DOI:** 10.1016/j.jbiomech.2015.06.021

**Published:** 2015-09-18

**Authors:** Richard A Hillam, Allen E Goodship, Tim M Skerry

**Affiliations:** University of Bristol, UK

**Keywords:** Bone strain, In vivo, Human, Tibia, Cranium

## Abstract

Bone mass and architecture are the result of a genetically determined baseline structure, modified by the effect of internal hormonal/biochemical regulators and the effect of mechanical loading. Bone strain is thought to drive a feedback mechanism to regulate bone formation and resorption to maintain an optimal, but not excessive mass and organisation of material at each skeletal location. Because every site in the skeleton has different functions, we have measured bone strains induced by physiological and more unusual activities, at two different sites, the tibia and cranium of a young human male in vivo. During the most vigorous activities, tibial strains were shown to exceed 0.2%, when ground reaction exceeded 5 times body weight. However in the skull the highest strains recorded were during heading a heavy medicine/exercise ball where parietal strains were up to 0.0192%. Interestingly parietal strains during more physiological activities were much lower, often below 0.01%. Strains during biting were not dependent upon bite force, but could be induced by facial contortions of similar appearance without contact between the teeth. Rates of strain change in the two sites were also very different, where peak tibial strain rate exceeded rate in the parietal bone by more than 5 fold. These findings suggest that the skull and tibia are subject to quite different regulatory influences, as strains that would be normal in the human skull would be likely to lead to profound bone loss by disuse in the long bones.

## 1 Introduction

Bone responds to loads it experiences, to grow and maintain a structure adequate for function, with safety factors for moderate overload (Currey, 1984; Lanyon, 1987). Excessive mass increases costs of growth, maintenance and use, so there are evolutionary reasons why tuned skeletons are advantageous over over-engineered ones. Consequences of failure on survival differ with skeletal site, so it is reasonable to assume that different bones have different safety factors.

The effect of loading bone is deformation or strain and for most long bones, deformations during peak physiological activities are 0.2–0.3% (Rubin and Lanyon, 1984). Some information related to bone strain during activity provides feedback for control of mass and architecture. Without high habitual strains, the effect of reduced usage, and specifically, removal of high magnitude/rate strain events leads to bone loss (Uhthoff and Jaworski, 1978; Skerry and Lanyon, 1995; Sugiyama et al., 2012). Bed rest, weightlessness or immobilisation in coma patients leads to profound reductions in whole body bone density (Whedon et al., 1976; Uhthoff and Jaworski, 1978; LeBlanc et al., 1987; Lavrijsen et al., 2007; Oppl et al., 2014). Similarly, post-menopausal changes are associated with bone loss and osteoporosis (Nordin et al., 1976).

However, there is one notable exception to loss of bone in the skeleton: the cranium of the skull does not lose bone during disuse or after menopause. In spaceflight, where bone is lost from limbs, there is increased cranial bone mass (Alexandre and Vico, 1996). Prolonged bed rest and spinal cord injury both result in bone loss from the appendicular and axial skeleton but not from the skull (Leblanc et al., 1990; Garland et al., 1992). Furthermore skull bones are resistant to postmenopausal bone loss (Gallagher et al., 1987) and osteoporotic fractures do not occur there (Kleerekoper and Avioli, 1993).

While animal studies show skull strains somewhat lower than limb bone strains (Ross and Metzger, 2004; Porro et al., 2014), there are no data on in vivo strains in the human skull, and only a few reports of human tibial strain recorded in vivo summarised in two recent review articles (Yang et al., 2011; Al Nazer et al., 2012), so we measured strains in the human tibia and cranium with simultaneous recording of bite and ground reaction forces. We show profound differences in physiological strain magnitudes and rates at the sites, suggesting either increased sensitivity to low strains in the skull, or insensitivity to effects of disuse.

## 2 Materials and methods

### 2.1 Strain gauges and subject

Three rosette strain gauges (EA-06-060RZ-120, Measurement Group, Basingstoke, UK) were prepared for implantation as described (Lanyon, 1973; Lanyon et al., 1975) and sterilised. The subject was a relatively normal healthy, 70 kg, 29 year old male (RAH). All procedures were approved by Bristol Healthcare Trust Ethical Committee to prevailing regulations and the subject gave consent for the work, and for his identity to be revealed. The gauges were applied, activities performed and gauges removed within one day.

#### 2.1.1 Surgical implantation of the strain gauges

The right tibia and scalp were prepared for aseptic surgery. Surgery was performed under bupivacaine anaesthesia (Marcain 0.25% with 1:200,000 adrenaline, Astra Pharmaceuticals Ltd., Hertfordshire, UK).

#### 2.1.2 Parietal bones

A 7 cm incision was made 2–3 cm lateral to the midline. Skin was retracted and haemorrhage controlled. A 1.5 cm square area of periosteum was elevated and bone scraped free of soft tissue. The bone was degreased with diethylether and chloroform. Cyanoacrylate adhesive (Histoacryl, Braun, Germany) was applied to the gauge. The gauge was placed onto the bone, so the central element was parallel to the sagittal plane. Pressure was applied for one minute to bond the gauge onto the bone. The incision was closed with lead wires passing out of the incision. The same procedure was performed on the contralateral bone. An image of a skull with a strain gauge attached in the same location and orientation is included in supplementary information.

#### 2.1.3 Tibia

Under identical local anaesthesia, a 7 cm incision was made parallel to the long axis of the tibia. The bone was prepared as before. The gauge was bonded with the central element parallel to the bone’s axis. This was confirmed by radiography. An image of a tibia with a gauge attached in the same location, and a photograph of a strain gauge in place is in Supplementary information.

### 2.2 Strain recording

Strain gauges were connected to amplifiers (2120A, Measurement Group, UK) whose output was fed into an A-D card (RTI-815, Analog, Norwood, USA) in a PC. Custom capture software was used (‘Super’, by D. McNally, University of Nottingham). Circuits were balanced with the subject relaxed (where bones were subjected to minimal stress) so that strain could be zeroed. Between sets of activities, gauges were re-zeroed. For the cranium, the relaxed position was achieved by sitting without conscious neck or face muscle activity and the head forward. For the tibia, the circuits were balanced with the subject sitting and the foot off the ground. Strains were recorded at 50 Hz for sedentary activities where no impact transients were expected and 500 Hz for more vigorous activities. No noise filtering was used

### 2.3 Bite force transducer

Measurements of bite force were made using a dental occlusal force meter, custom-made by staff at the Medical Physics Department, Sunderland General Hospital, UK. The device incorporated a 1000 N loadcell between dental occlusal pads. Load cell output was connected to the same amplifiers and PC as used for capture of strain data to allow simultaneous recordings. Calibration was performed by hanging known masses on it.

### 2.4 Ground reaction force measurements

We made force plate recordings (Kistler Instruments, Winterthur), as described previously (Dow et al., 1991; Williams et al., 1999). Output from the force plate was converted and stored as for strain, then processed to calculate the orthogonal ground reaction force parameters using in-house software, exported to Microsoft Excel for analysis and Graphpad Prism for display. For technical reasons we were unable to record GRF during walking and squat exercises. The figures display only Fz, but all GRF recordings are in supplementary information.

### 2.5 Activities

A range of activities were planned.

1. Biting onto a dental occlusal force meter with cheek teeth and incisors.
2. Eating a banana.
3. Grimacing/pulling facial expressions.
4. Walking at ~0.7 m s^−^^1^.
5. Performing squats with a 25 kg weight.
6. Jumping from 0.45 m.
7. Heading a 4.5 kg medicine/exercise ball.
8. Jumping from 1.3 m.

Except for the 1.3 m jump, all activities were performed with the feet protected by socks. For the 1.3 m jump, rubber soled, leather boots (Trader, Debenhams, UK) were worn.

### 2.6 Data analysis

Principal strain magnitudes were calculated from individual strain element data. Strain rates were calculated by selecting maximum rise in strain for each activity, calculated over 40 ms periods. The start time of the 40 ms period is given in results. The full set of recorded bite force, ground reaction force and strain data (and derived strain rate data) are in Supplementary information. Because the experiment had only one subject, we selected representative traces for each activity to display in figures, and we have not undertaken statistical analysis.

## 3 Results

### 3.1 Strain measurements

Where relevant, we made simultaneous recordings of parietal and tibial strains. For activities that involved no significant body movement, we recorded only parietal strains, as tibial strains were unrelated to activity. The right parietal gauge and the tibial gauge functioned well and we recorded credible strains from them. The central element of the left parietal gauge did not produce any data other than low level noise and we excluded data from that gauge in results presented. However, raw strain magnitudes from the functioning elements of that gauge were comparable with those from the right parietal gauge. The right parietal gauge worked it was detached following a direct impact from heading the medicine ball. The time this occurred was obvious because of immediate formation of a large haematoma and subsequent strain recordings that were quite different from earlier recordings. After this, parietal data were excluded. The only remaining activity to be performed was the jump from 1.3 m, so we have only tibial strain data for that. The tibial gauge functioned throughout the experiment. We recorded strains (and bite force/ground reactions) for at least 3 repetitions of each activity.

### 3.2 Strains during activities

#### 3.2.1 Activities inducing parietal strain

**Fig. 1:**
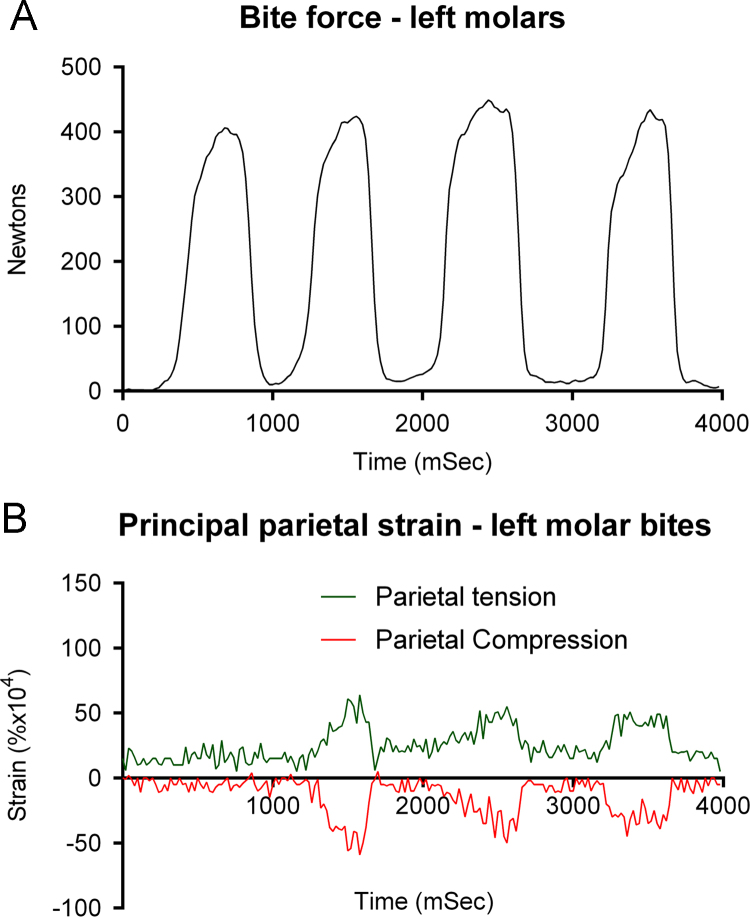
Simultaneous recordings of left molar bite force and parietal principal strain, where the subject grimaced during the bites.

Bite force and strain were recorded with the bite force transducer placed between molar teeth and incisors. Biting was associated in some but not all cases with apparently related increases in parietal strain (Fig. 1A and B), where peak principal compressive and tensile strains recorded were +0.0064% and −0.0059% at left molar bite force of 420 N. In the same recording, at slightly higher peak molar bite force of 449 N, strains were +0.0043% and −0.0014%, respectively. Peak parietal strain rate was 0.925% s^−1^ at 1640 ms, when bite force was being released.

**Fig. 2:**
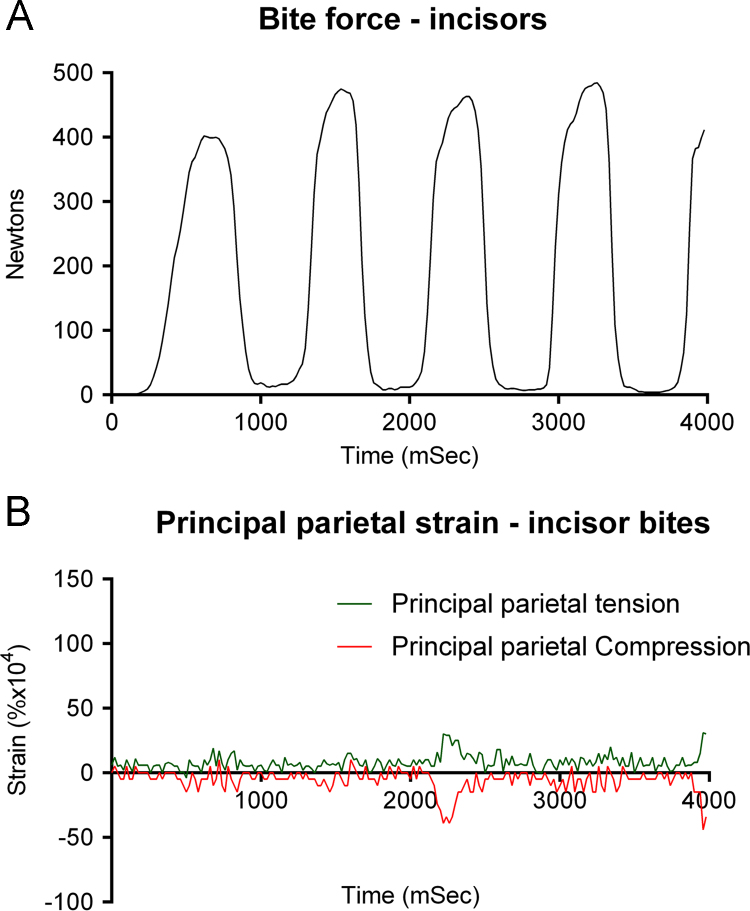
Simultaneous recordings of incisor bite force and parietal principal strain where the subject maintained a nearly constant facial expression during the bites.

Other recordings revealed no relationship between bite force and parietal strain, and what appeared to be little more than noise in traces. For example, a bite with incisors of 430 N was associated with peak principal compressive and tensile strains of +0.0029% and −0.0039% (Fig. 2A and B), although other bites had similar magnitude of force, but generated strains well below 0.002% in both tension and compression. In general, there was no consistent difference between biting with cheek teeth and incisors, and low strains were recorded for both, depending on facial expressions. Peak incisor bite parietal strain rate was 0.625% s^−^^1^ at 720 ms, during a bite on the transducer.

**Fig. 3:**
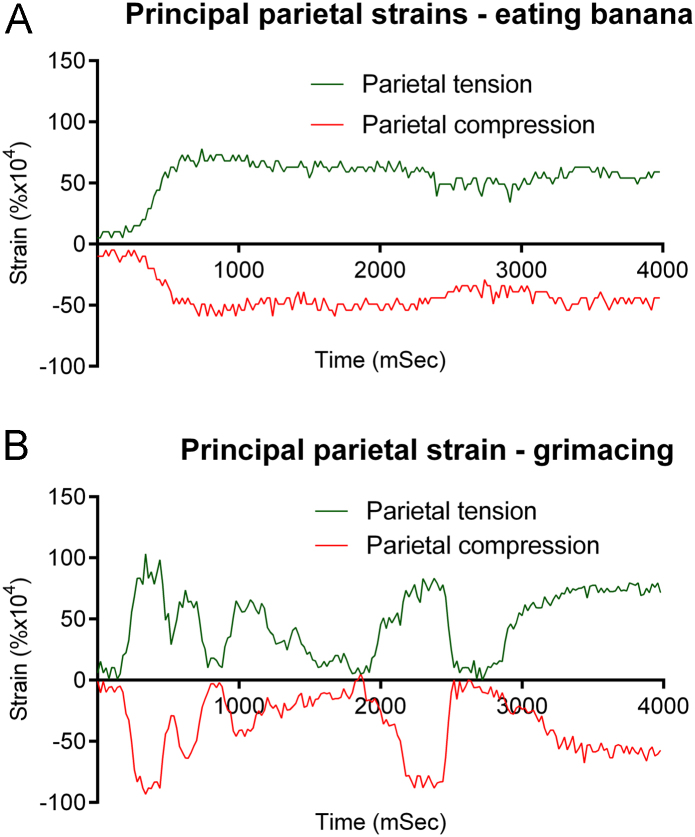
Recordings of parietal principal strain while biting then chewing a banana (A) and during grimacing or pulling a face (B).

Recordings of parietal strains during other activities supported the role of facial contortions/movements rather than bite force, in inducing parietal strains. Eating a banana was associated with peak parietal compressive and tensile strains of +0.0078% and −0.0059%, respectively (Fig. 3A), which exceeded strains associated with biting hard. Peak parietal strain rate was 0.375% s^−1^ many times during the recording.

However it was unnecessary to exert force with jaw muscles closing teeth together to induce parietal strains of similar magnitude. Simply grimacing induced peak parietal principal compressive and tensile strains of +0.0103% and −0.0093% (Fig. 3B), which exceeded strains associated with biting hard or chewing. Peak parietal strain rate was 1.322% s^−1^ at 2400 ms, as the subject relaxed expression quickly.

#### 3.2.2 Physiologically relevant activities inducing strains at both sites

##### 3.2.2.1 Walking

**Fig. 4:**
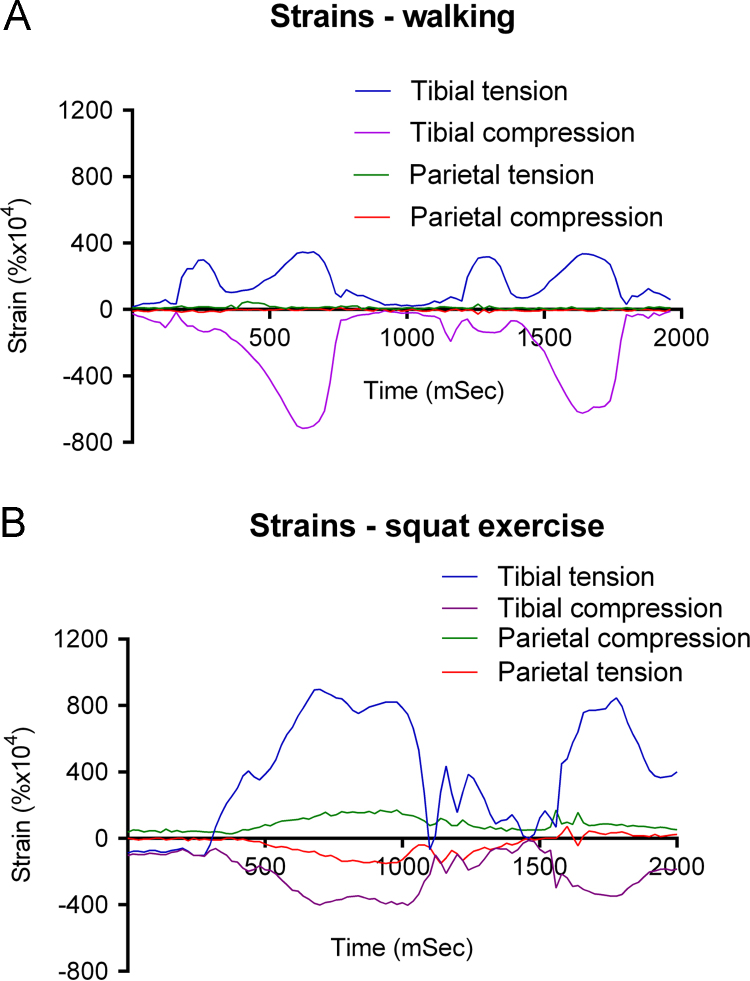
Simultaneous tibial and parietal principal strains during 2 strides of walking (A) and 2 cycles of a squat exercise where the subject held a 25 kg weight bar squatting position then stood up vigorously before squatting again (B).

A typical recording during walking at approximately 1 Hz and 0.7 m s^−1^ shows heel strike at around 200 ms, pushoff at about 580 ms, and heel strike again at about 1200 ms. During these events, we found that tibial strains rose rapidly to +0.0318% and −0.0139% at heel strike (Fig. 4A). There were larger (but slower rate) compressive and tensile peaks of +0.0341% and −0.0712% at pushoff. Peak parietal compressive and tensile strains were +0.0047% and −0.0027%, but these maxima were unrelated to gait phase, occurring at different places in successive strides. Peak tibial strain rate was 10.144% s^−1^ at 700 ms, just before pushoff. The peak parietal strain rate was 1.026% s^−1^ at 380 ms (heel strike).

##### 3.2.2.2 Squat exercises

More vigorous activity induced higher tibial strains. Squat exercises with a 25 kg weight bar were associated with peak tibial principal compressive and tensile strains of +0.0898% and −0.0402% (Fig. 4B). At the same time principal parietal strains were +0.0130% and −0.0078%. Peak principal parietal strains were +0.0171% and −0.0151%, but later during recording, at the end of the squatting phase, when tibial strains were less than maximal values but still over +0.08 and −0.035. Peak tibial strain rate was 15% s^−1^ at 1060 ms as the subject squatted, lowering tibial strains quickly. Peak parietal strain rate was 2.925% s^−^^1^ at 1600 ms before another rise to standing. The subject did alter facial expression during these actions due to effort needed to perform them. He was unable to perform squats without changing his facial expressions.

##### 3.2.2.3 Jumping from 0.45 m

**Fig. 5:**
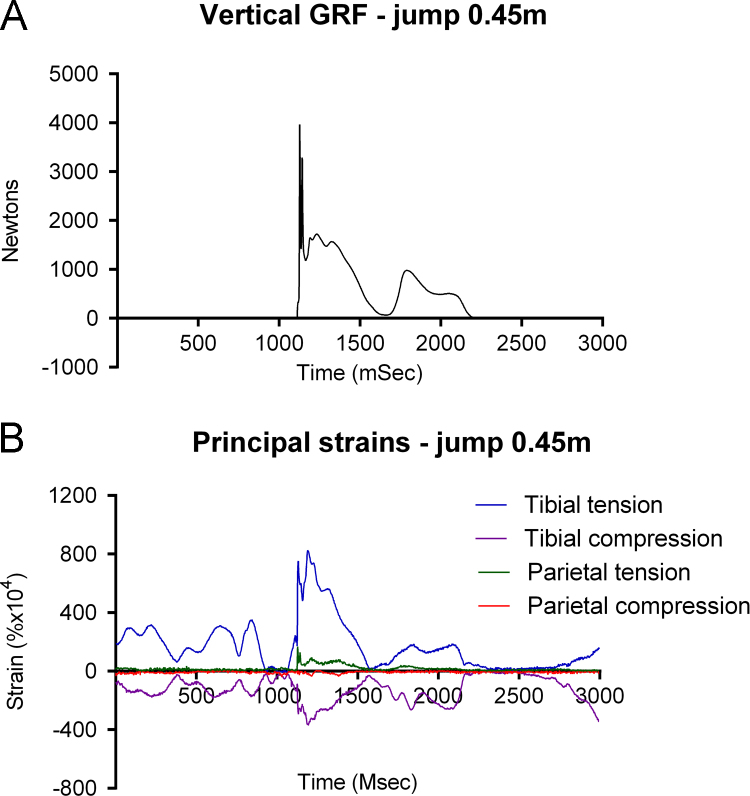
Simultaneous recording of vertical ground reaction force (A) and tibial and parietal principal strains (B) while preparing for and then jumping from a height of 0.45 m onto a force plate.

When the subject jumped from 0.45 m onto the force plate, he landed (at 1130 ms), with momentary loss of balance causing reduction in GRF (1630 ms), and recovered (1800 ms), then stepped off the plate at 2170 ms (Fig. 5A). We recorded a series of principal strains of between +0.03 and +0.04% and −0.015 and 0.018% from the tibia just before jumping, but parietal strains below +0.0025% and −0.0015% that were unrelated to tibial peaks (Fig. 5B). As the subject jumped, tibial strains fell briefly to near zero, though at the same time parietal strains were unchanged. At landing, tibial principal strains rose to +0.082% and −0.0366%, when we recorded parietal principal strains of +0.0171% and −0.0025%. At landing, vertical GRF (Fz) peaked at 3,967 N, 5–6 times bodyweight. Peak tibial strain rate was 15.35% s^−1^. Peak parietal strain rate was 3.925% s^−1^. Both strain rate peaks coincided with landing.

#### 3.2.3 Activities with no normal physiological counterparts

**Fig. 6:**
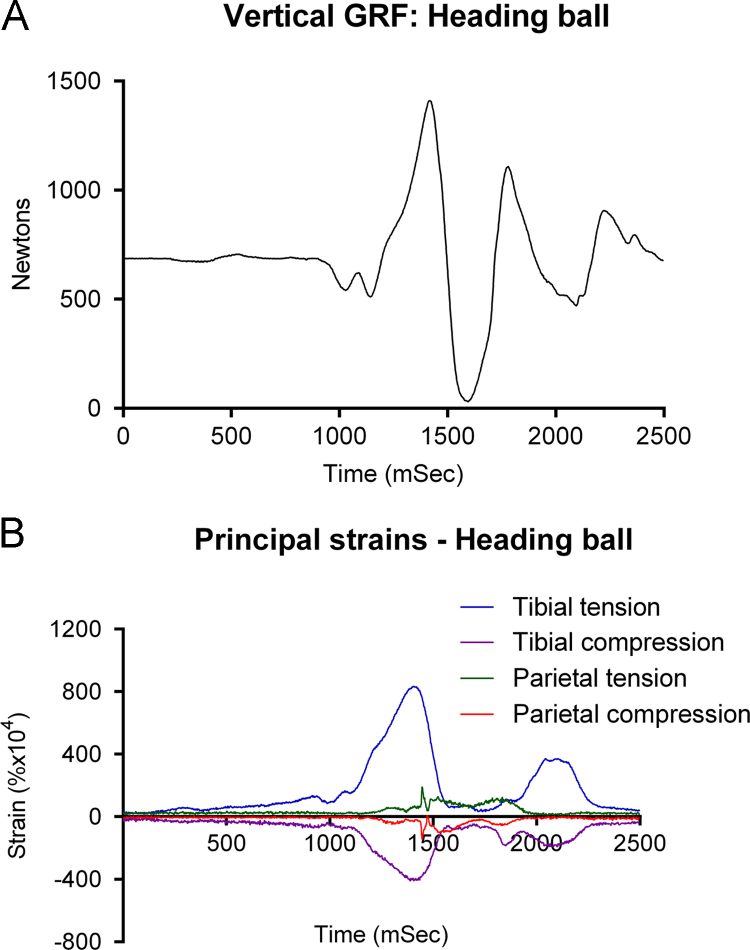
Simultaneous recording of vertical ground reaction force (A) and tibial and parietal principal strains (B) while jumping to head a 4.5 kg medicine ball. Impact occurs while the subject is airborne just after peak GRF and tibial strains.

To determine the influence of more extreme events, the subject agreed to perform activities for which he would be unprepared. He headed an ordinary football and then, without notification for the first time, a medicine/exercise ball of similar dimensions, weighing 4.5 kg. Vertical ground reaction force (Fz) rose from body weight (690 N) to 1421 N at 1422 ms, as he jumped to head the ball, leaving the ground briefly (Fig. 6A). Peak principal tibial strains at 1400 ms rose to +0.083% and −0.0402% (Fig. 6B). Peak principal parietal strains occurred 40 ms after peak GRF and tibial strain, while the subject was jumping upwards, with values rising to +0.0192% and −0.0142%. Because there was no change in GRF or tibial strain at peak parietal strain, we believe impact with the ball occurred while the subject was airborne. Peak tibial strain rate was 7.675% s^−1^ at 1470 ms during landing. Peak parietal strain rate of 3.225% s^−1^ was recorded between 1406 and 1446 ms, at impact.

**Fig. 7:**
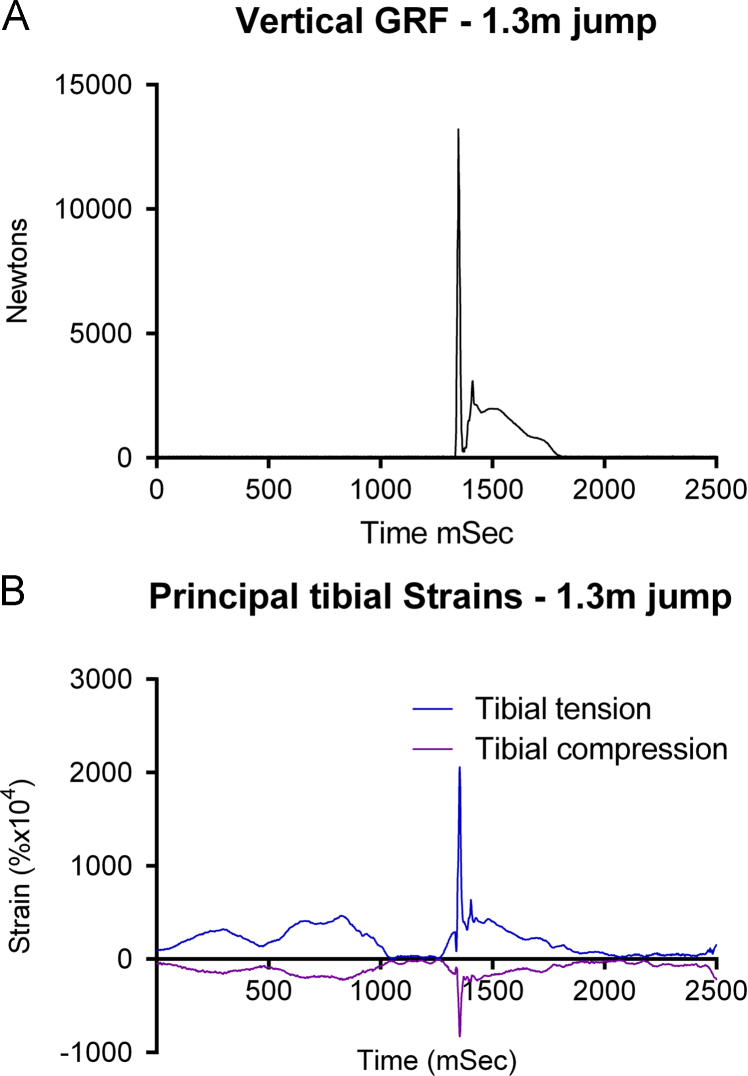
Simultaneous recording of vertical ground reaction force (A) and tibial principal strains (B) while preparing for and then jumping from a height of 1.3 m onto a force plate.

The final event was that the subject jumped from 1.3 m onto the force plate. Unfortunately the medicine ball had struck the right parietal gauge site and dislodged it, so only meaningful tibial strains and ground reaction forces were recorded. Ground reaction force rose to a peak of 13,242 N at 1348 ms (Fig. 7A), with peak tibial strains of +0.2066% and −0.0836% occurring 6 ms later (Fig. 7B). Peak tibial strain rate was 45.525% s^−^^1^ at approximately 1310 ms.

Maximum strain magnitudes and rates presented in Table 1.

## 4 Discussion

The overall finding from these studies is that there are clear differences between strain magnitudes and rates experienced by the cranium and the tibia in a living human. If we separate activities into those that might be experienced physiologically (even unusually) during daily activity and those which would be very rare events – verging on incidents likely to cause trauma – we can compare effects of walking and squats, and chewing, grimacing and biting separately from heading the medicine ball and jumps from 0.45 and 1.3 m. Under physiological circumstances, both peak strain magnitudes and strain rates are much lower in the cranium than the tibia. Surprisingly, highest physiological parietal strains (+0.0171 and −0.0151%) were recorded during squat exercises, where there was no apparent relationship with phase of the exercise, GRF or tibial strains. This was explained by analysis of data from recordings of parietal strain during biting, eating and grimacing. Those show that strains recorded during maximal biting force were less than during eating the banana and less again than strains during forceful grimacing. During bites when the subject tried to maintain an even expression while biting the transducer, strains were much lower (Fig. 2). The ability of the subject to induce significant parietal strains without biting hard or even closing the teeth together was a surprise. Taken together, these features of our data provide compelling evidence that peak physiological parietal strain in humans is generated primarily by contraction of the muscles of facial expression, not biting. Had we recorded video of the subject during squat exercises we might have been linked those high parietal strains with facial contortions.

More unusual activities – jumping from 0.45 and 1.3 m for the tibia, and for the skull, heading the medicine ball also reveal clear differences between the sites. Heading the medicine ball was associated with highest peak parietal strain magnitudes of all activities, but they were hardly different from expression-induced strains during squats (0.19% and 0.17% respectively), and less than double those induced by grimacing. In the tibia, peak strain magnitudes during landing from a jump from 1.3 m (+0.2 and −0.08%), were reasonably consistent with peak principal tibial strains in the other studies of human bone strain in vivo during vigorous activity (Yang et al., 2011). The difference between strains induced by unusual events was greater than 10 fold between the sites. As it has been suggested that unusual error strains are the predominant drivers for adaptation, that difference between the sites could be of functional significance.

Data on strain rate are interesting, but we have to be cautious in interpreting them. As the parietal strains are small, the signal to noise ratio is low compared with the tibial data. This means that over brief periods of time, the rates we calculated could be influenced by noise and might be over-estimates of the real rates. We chose this very short time of 40 ms for the rate analysis because we wished to make our analysis as critical as possible and likely to show no difference between the two sites. We made test comparisons of strain rate calculations using smoothed data, or averaging at a 3–10 fold lower resolution of data, and in every case, the difference in strain rate between the two sites was greater than we present here. We therefore feel that the >5-fold lower physiological strain rate we see in the parietal bone represents a very cautious but real difference between the two sites. We include the raw principal strain rate data in the Supplementary information in order that others with more expertise in strain data analysis can explore the issue further. The specific findings we highlight are interesting – the highest physiological tibial strain rate was 15% s^−1^, over 5 times the highest physiological parietal rate of 2.925% s^−1^. In more unusual activities, peak rates in both sites were during the 0.45 m jump, where the tibial rate of 15.35% s^−1^, was over 3 times higher than peak rate in the skull during the same jump (3.95 s^−^^1^). It is unfortunate that we could not acquire strain data from the skull during the 1.3 m jump, because higher parietal magnitudes and rates might have been recorded. If the noise in the data affected the strain rate calculations, then this difference could be greater than we suggest.

Naturally, the major limitation to this study is the use of one subject and only a single 3 element strain gauge on each bone. We are unable to use valid statistical methods to determine the significance of differences between the two sites, given that limitation. However, the first recording of human bone strain was in a single subject (Lanyon et al., 1975) and was followed by larger numbers in later studies which confirmed the findings of that first report (Burr et al., 1996; Foldhazy et al., 2005; Yang et al., 2011). Because we use well-established techniques, we are confident that data we show here represent a good approximation of differences between the sites.

Cranial strains have not been recorded from a living human before so we can only compare our data with strains from animals. In such studies, it is clear that differences between long bone and cranial strains exist in rats (Rawlinson et al., 1995). In pigs, cranial strains during physiological activities are low, although electrical stimulation of the masseter muscle has been shown to induce strains in the maxilla >0.08% (Herring et al., 1996; Herring and Teng, 2000). In parts of the skull high strains are experienced. Strains of ~0.2% have been recorded from zygomatic arches of primates biting hard foods (Hylander and Johnson, 1997), while strains over 0.1% have been recorded from mandibles of the hyrax (Lieberman et al., 2004). However those data are not from the site where we recorded skull strains and the function of them is much more likely to reflect significant mechanical functions during activity. It is also possible that inability to induce animals to pull faces without biting or chewing limits valid comparisons with our study.

Our data lead to a conclusion that one of two possible biological explanations accounts for differences between the sites. It is possible that cells in the cranium are sensitive to effects of strains that would be regarded as disuse in long bones, and skull mass and architecture is subject to the same adaptive mechanisms as the rest of the skeleton. Alternatively, the skull may be insensitive to lack of significant strain-related stimulus. The first possibility appears unlikely given the lack of bone loss in many circumstances where bone is lost from the rest of the skeleton but not the skull. If the skull simply had a low setpoint, we would expect bone loss in the skull with disuse or hormonal changes when there is none. It is clear that bones develop a genetically determined shape and architecture that is usually modified by prevailing mechanical environment (Lanyon, 1980; Gomez et al., 2007). However, there is no reason to suppose that the cranium should not be at genetic baseline un-modified by significant loading-related effects. The reason for such a mechanism would be explained by consideration of the cost of failure of bones at the sites. We can hypothesise that adapted or tuned skeletons conferred increased ability to survive, compared with over-engineered skeletons because of issues of efficiency of locomotion and evasion from predators/capture of prey. Cost of failure of long bones (fracture) would usually be catastrophic, but could under some circumstances be survivable. However cost of failure of the skull with consequent brain damage would be much less likely to be survivable. That idea can be linked to the data we have recorded here. If we compare the ratio of strain to failure (~0.8%) to peak physiological strain, we can calculate a safety factor for different bones. Here, the safety factor for the tibia is 3 or 4:1 but in the skull over 40:1.

One implication of this finding is in development of novel targets for therapies for bone diseases. Identification of mechanisms that underlie different responses of cells at the two sites could show target pathways that would make long bone cells behave more like skull cells despite low or absent strains, resistant to bone loss. Studies on explants or cells isolated and cultured ex-vivo from the two sites in rodents reveal few differences (Rawlinson et al., 1995; Kingsmill et al., 2013), and it is possible that site specificity of response is due to both cell and matrix contributions to interaction between the cells and their physiological environment. A second implication of this work is that we believe it overturns the idea that Harold Frost’s “mechanostat” can be represented as a general numerical value for the skeleton (Frost, 1987). Instead we propose that the skeleton has a spectrum of different set points which relate to habitual use, importance to survival, and likelihood of damage. In long bones that set point may be in the order of 0.15%, but in the skull it is so much lower that genetic influences dominate over functional ones.

## Conflict of interest statement

None of the authors have any conflicts of interest.

## Figures and Tables

**Table 1:** Summary of peak principal strain magnitudes and rates. (Summary of strain magnitudes recorded from the tibia and skull.)

**Activity**	**Max principal tensile strain % (E1)**		**Max principal compressive strain % (E2)**		**Max principal strain rate (%/s)**	
	**Skull**	**Tibia**	**Skull**	**Tibia**	**Skull**	**Tibia**

**Biting (molars)**	0.0064		0.0059		0.925	
**Biting (incisors)**	0.0029		0.0039		0.625	
**Eating banana**	0.0078		0.0059		0.375	
**Grimacing**	0.0103		0.0093		1.322	
**Walking**	0.0047	0.0341	0.0027	0.0712	1.026	10.144
**Squat+weight**	0.0171	0.0898	0.0151	0.0402	2.925	15.0
**Jump (0.45 m)**	0.017	0.082	0.0025	0.0366	3.925	15.35
**Heading ball**	0.0192	0.084	0.0142	0.0402	3.225	7.675
**Jump (1.3 m)**	–	0.2066		0.0836	–	45.525
